# Duplicated pituitary gland plus syndrome with thoracoabdominal findings suggesting abnormal axial mesodermal signaling and ciliopathy

**DOI:** 10.1007/s00247-025-06281-8

**Published:** 2025-06-07

**Authors:** Asha Sarma, Lindsey A. Knake, Marta Hernanz-Schulman, Thomas Cassini, Sumit Pruthi

**Affiliations:** 1 Division of Pediatric Imaging, Vanderbilt University Medical Center, 2200 Children’s Way, Nashville, TN 37232 United States; 2 University of Iowa, Iowa City, United States

**Keywords:** Ciliopathy, Duplicated pituitary gland plus syndrome, Brain malformations

## Abstract

Duplication of the pituitary gland is a rare anomaly with variable associated craniofacial malformations (duplicated pituitary gland plus syndrome). Thus far, malformations have only been reported in the craniofacial structures, central nervous system (CNS), and spine. This report illustrates a severe case with additional, previously unreported body features including anomalies of the lungs, heart, liver, spleen, and abdominal vasculature. A description of this case will aid in comprehensive diagnosis of anomalies in patients with duplicated pituitary gland plus syndrome. Moreover, this case may improve understanding of the etiology of this rare disorder and its embryological underpinnings.

## Introduction

Duplicated pituitary gland is a rare disorder that may be isolated or occur in conjunction with variable associated brain and craniofacial malformations (“duplicated pituitary gland plus syndrome”). Fewer than 70 cases have been described in the literature [1]. Among the most common associated brain anomalies are “prominence of the hypothalamic midline” (referred to in the literature by various terms, including “tuberomammillary fusion”); callosal anomalies (e.g., dysgenesis, pericallosal lipoma); arterial anomalies (e.g., short or non-fused basilar arteries); brainstem and upper cervical spinal cord anomalies (e.g., cervical and medullary “limited ventral myeloschisis,” or anterior tethering bands of the medulla or upper cervical spinal cord); brainstem patterning defects and “butterfly” appearance of the medulla; olfactory bulb hypoplasia or aplasia; and temporal lobe anomalies (e.g., temporal cortical clefts, hippocampal malrotation) [1]. Osseous anomalies affect the skull base, face, and vertebrae, including persistent craniopharyngeal canal, sellar widening or duplication, cleft lip and palate, bifid tongue and uvula, midline oropharyngeal mass (e.g., teratoma), and vertebral anomalies including “zipper-like” ventral clefting [1]. This case includes a new description of thoracoabdominal manifestations, including horseshoe extralobar pulmonary sequestration, double outlet right ventricle, accessory heterotopic splenic tissue, midline liver, and duplicated infrarenal inferior vena cavae. This description may aid clinicians in planning comprehensive diagnostic evaluation of patients with duplicated pituitary gland plus syndrome. Furthermore, it may contribute to elucidation of the etiopathogenesis of this complex syndrome.

## Case report

**Fig. 1:**
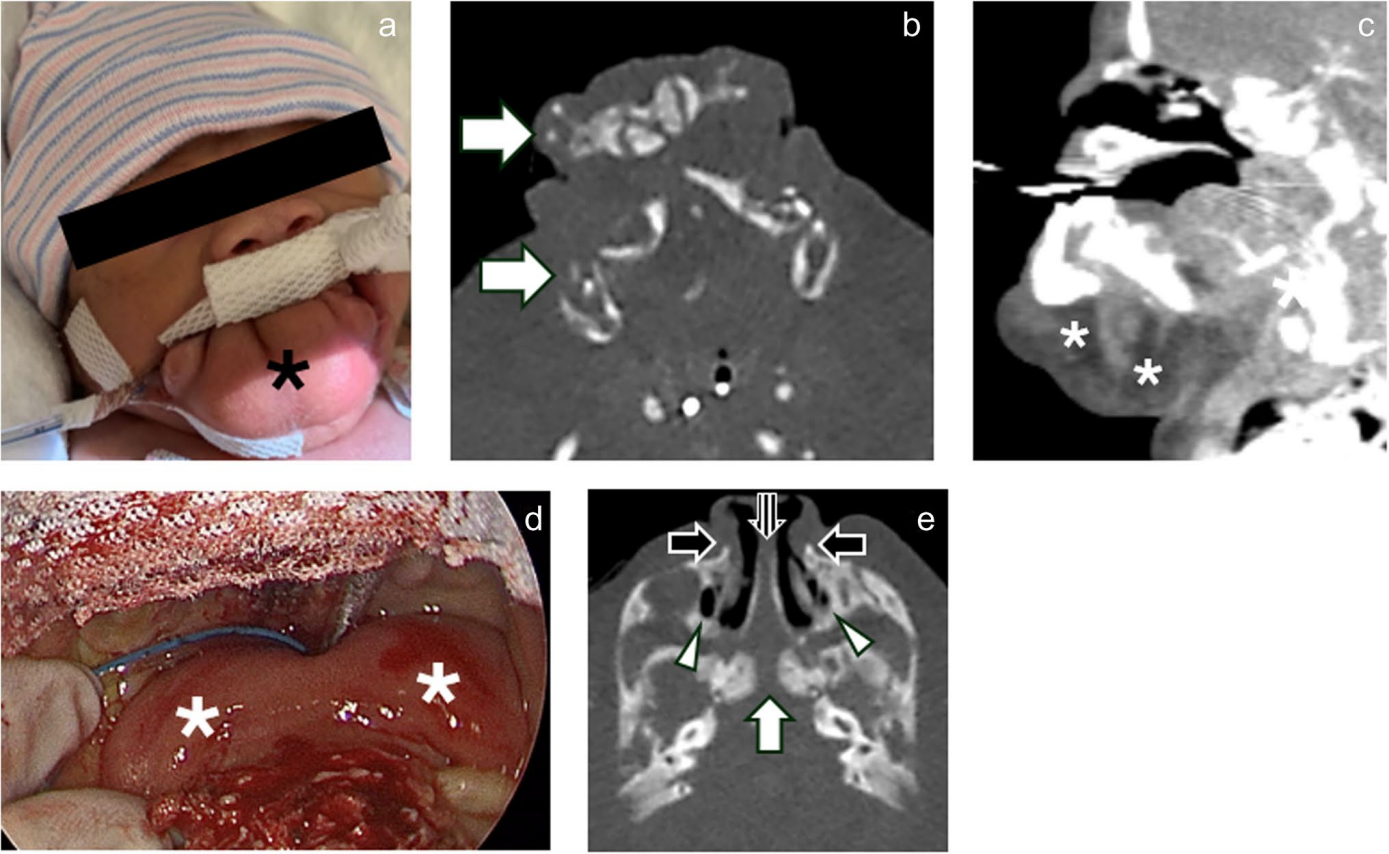
Craniofacial malformations. Clinical image (**a**) and axial (**b**) and sagittal (**c**) images of the face from a head CT angiogram demonstrate a skin-covered orofacial mass-like prominence (*black asterisk* in **a**) containing jaw-like elements that were anteriorly displaced from the dysplastic mandible (*arrows* in **b**) and excessive fat and soft tissue (*white asterisks* in **c**). Laryngoscopic image of the oral cavity (**d**) shows a bifid tongue (*white asterisks* in **d**). Axial oblique (**e**) image of the face and central skull base demonstrates a widened sella with a central osseous cleft (*white arrow*). Closed duplicated craniopharyngeal canals were associated with each of two pituitary fossae which were located parasagittally (*not pictured*). In addition, there is a widened pyriform aperture (*black arrows*), and duplication of the vomer (*striped arrow*) with widening of the nasal cavity. Each choana was duplicated, and the lateral choanal openings appeared blind ending (*arrowheads*). Laryngoscopic image is courtesy of Dr. Amy Whigham.

A male infant was born by C-section to a 30-year-old G1P1 mother at 31 weeks gestational age. Preterm delivery was due to preterm labor and poor fetal heart tracing. The patient was intubated in the delivery room due to severe airway obstruction by a skin-covered orofacial mass-like prominence that had been diagnosed prenatally along with other anomalies. Clinical examination also demonstrated a bifid tongue (Fig. 1). Maxillofacial CT demonstrated a widened pyriform aperture and nasal cavity with duplicated vomer and choanae (Fig. 1) and helped to further characterize the abnormal orofacial tissue containing disorganized mandibular elements and fibrofatty components. Upon resection, histopathologic examination showed “duplicated maxillary complex versus mesenchymal hamartoma.”

**Fig. 2:**
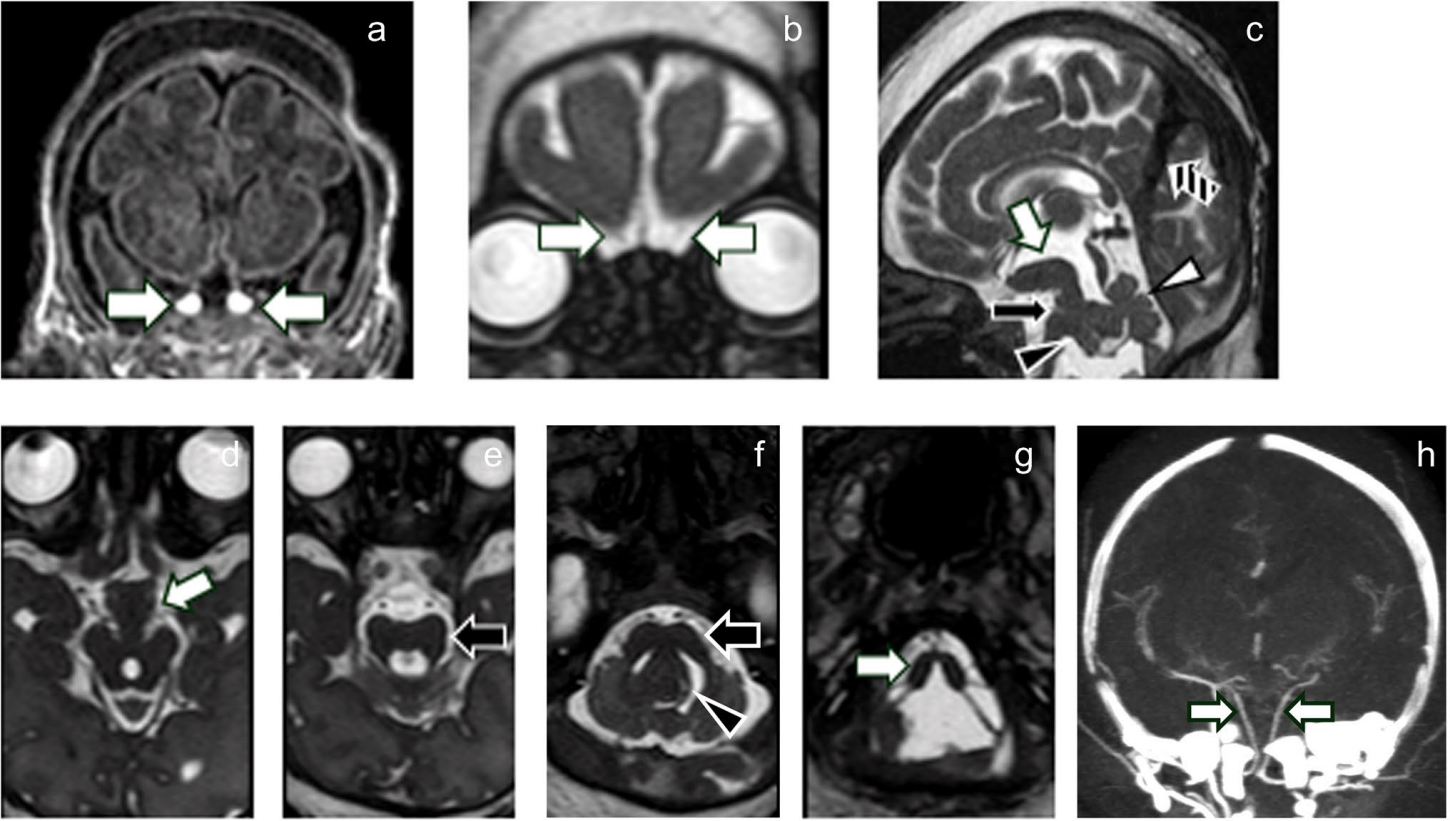
Intracranial malformations. Coronal T1-weighted MRI (**a**) demonstrates duplication of the pituitary gland and infundibulum (*arrows* in **a**). Coronal balanced fast-field echo T2-weighted MRI (**b**) demonstrates marked olfactory bulb hypoplasia or aplasia (*arrows*). Sagittal (**c**) and axial (superior to inferior, **d**-**g**) balanced fast-field echo T2-weighted MRI demonstrates a “prominent hypothalamic midline” and diencephalic-mesencephalic junction abnormality (incomplete separation of the diencephalic tissue and midbrain; *white arrows* in **c** and **d**). There is an unusually severe hindbrain malformation including a widened pons with narrow AP diameter (*black arrows* in **c**, **e**, and **f**) and subtle ventral clefting (*black arrow* in **c**), cerebellar hemispheric hypoplasia, vermian hypodysplasia with increased tegmentovermian angle (*white arrowhead* in **c**), dysplastic tissue resembling the cerebellum that is inseparable from the dorsal pons and lateral aspect of the fourth ventricle (*black arrowheads* in **c** and **f**), small middle cerebellar peduncles, and an abnormal-appearing medulla with a deep dorsal cleft (*white arrow* in **g**). There is a persistent falcine sinus (*striped arrow* in **c**). Non-fused paired vertebral arteries are seen on a coronal CT angiogram maximum intensity projection image (**h**).

Craniospinal MRI demonstrated a widened, partially duplicated sella with duplicated pituitary gland and stalk, “prominence of the hypothalamic midline,” diencephalic-mesencephalic junction abnormality, and an unusual, severe hindbrain malformation involving both the brainstem and cerebellum (Fig. 2) [1]. CT angiography of the head showed paired, non-fused basilar arteries. In addition, there was a persistent falcine sinus (Fig. 2). The olfactory bulbs were aplastic or markedly hypoplastic (Fig. 2). No other supratentorial abnormalities were identified.

**Fig. 3:**
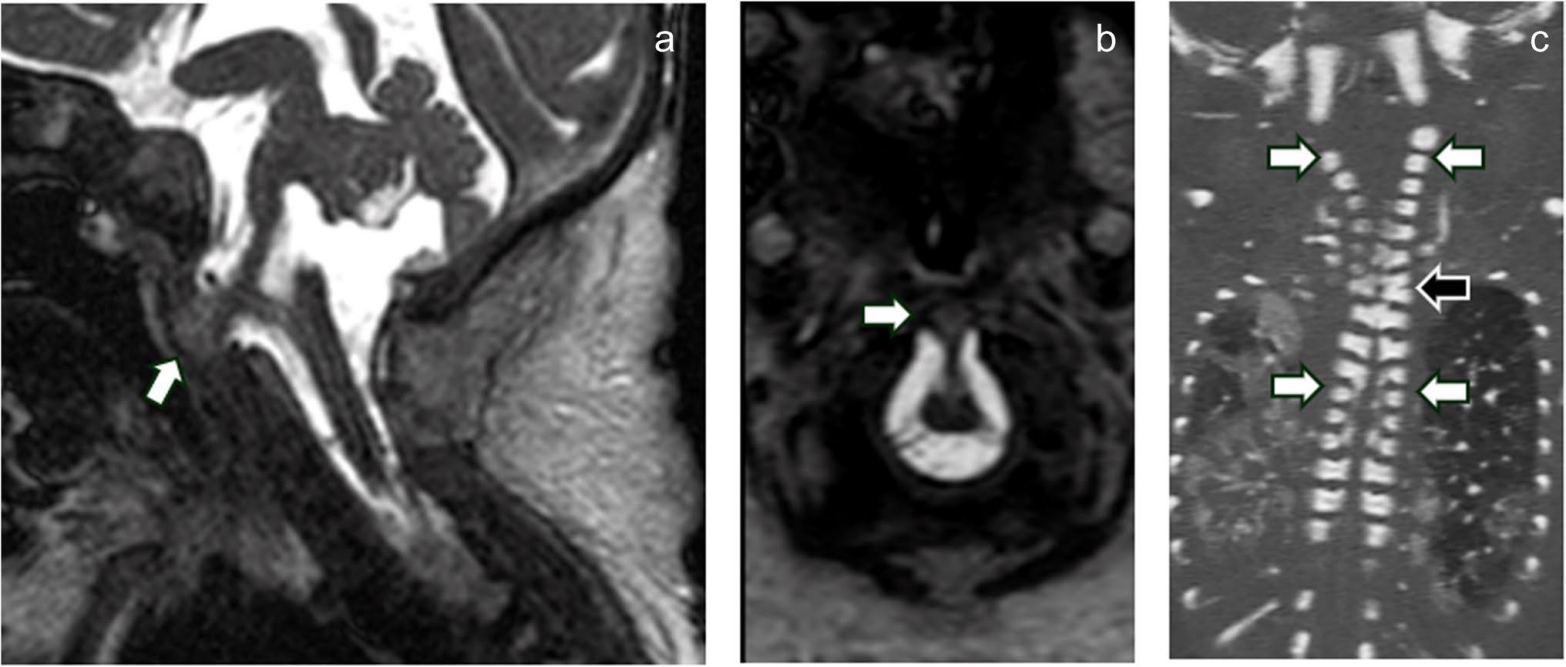
Spinal malformations. Sagittal (**a**) and axial (**b**) balanced fast-field echo T2-weighted MRI demonstrates “limited ventral myeloschisis” (*arrows* in **a** and **b**). Coronal oblique CT of the cervical and thoracic spine (**c**) demonstrates “zipper-like” anterior vertebral clefting (*white arrows*) and a vertebral formation and segmentation anomaly at C6-C7 (*black arrow*)

Spinal imaging demonstrated an upper cervical anterior vertebral cleft with “limited ventral myeloschisis” (Fig. 3). There were also multilevel “zipper-like” cervical and thoracic anterior vertebral body clefts and cervicothoracic vertebral anomalies (Fig. 3) [1].

**Fig. 4:**
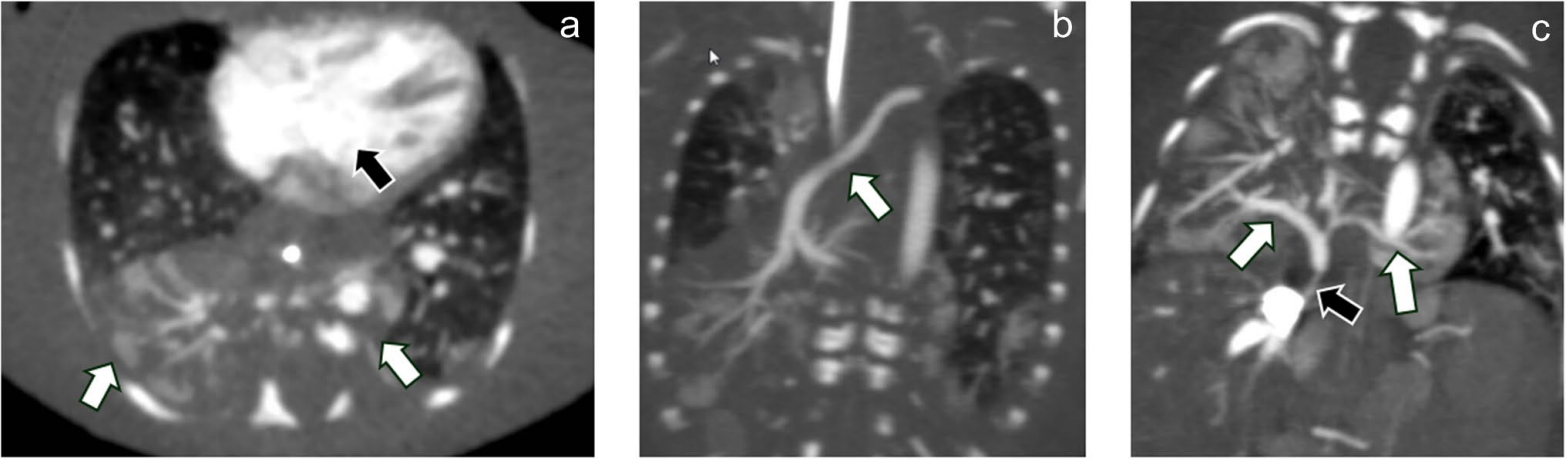
Thoracic malformations. Axial (**a**) and coronal (**b**, **c**) CT angiographic images of the chest demonstrate an extralobar sequestration with a “horseshoe” configuration centered in the right lung base, crossing midline (*white arrows* in **a**). Note how the blood vessels within the sequestered lung cross midline. A dominant feeding artery (*arrow* in **b**) originates from the dorsal thoracic aorta near the left subclavian artery origin, taking an oblique supero-inferior mediastinal course to the right base. Draining veins (*white arrows* in **c**) empty into the inferior vena cava through a narrow channel (*black arrow* in **c**). There is a ventricular septal defect in this patient with double outlet right ventricle (*black arrow* in **a**)

**Fig. 5:**
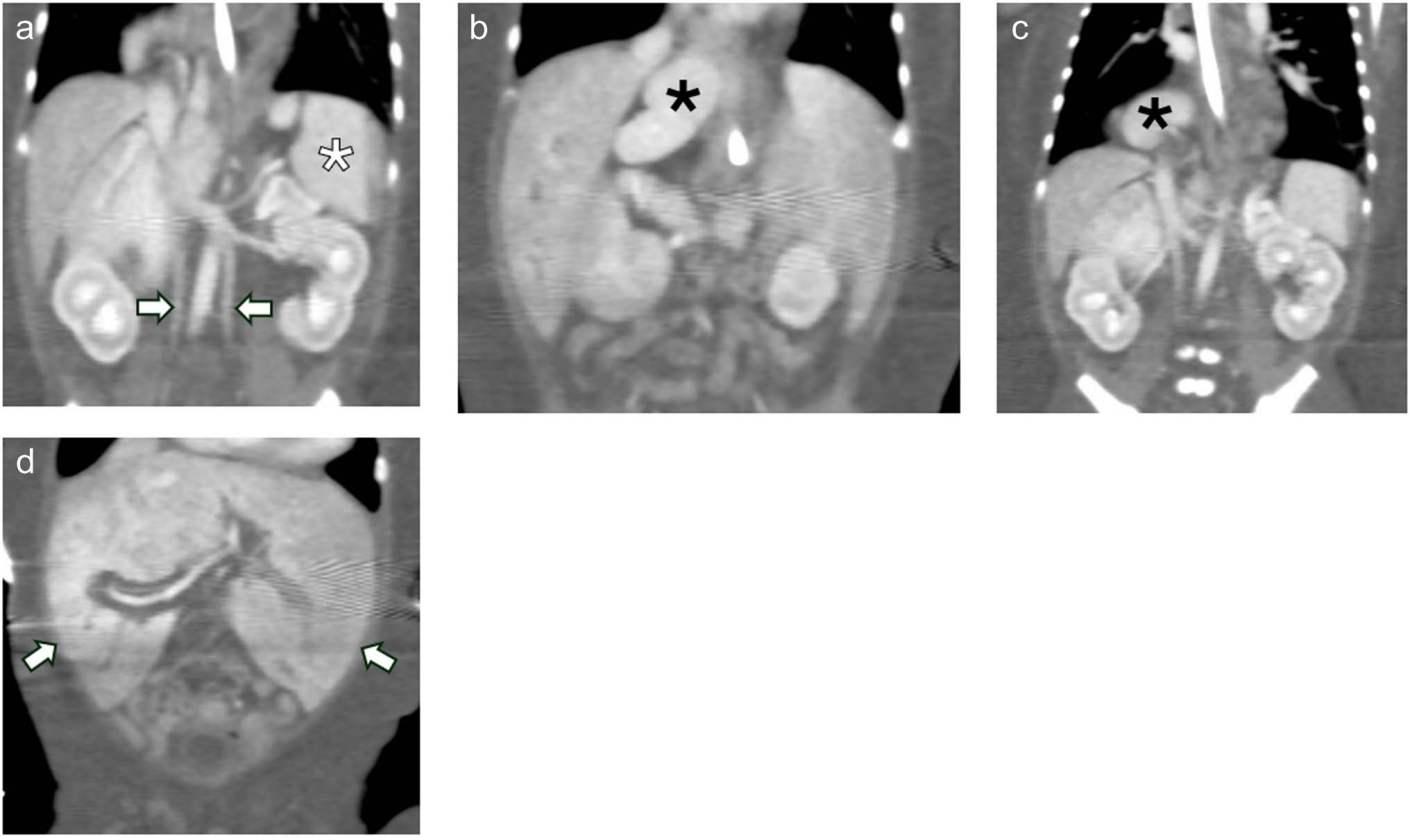
Abdominal malformations. Coronal CECT of the abdomen (**a**-**c**) demonstrates an orthotopic spleen (*white asterisk* in **a**) and unusually located heterotopic splenic tissue in the right upper quadrant and thorax (a pattern not observed in typical polysplenia) (*black asterisks* in **b** and **c**) and duplicated infrarenal inferior vena cavae (*arrows* in **a**). Coronal CECT of the abdomen also demonstrates a symmetric, bifid appearing liver (*white arrows* in **d**)

Thoracic anomalies included double outlet right ventricle with a ventricular septal defect, extralobar pulmonary sequestration with a “horseshoe” configuration spanning midline (Fig. 4), and agenetic right upper lobe bronchus. Abdominal anomalies included heterotopic accessory splenic tissue in the right chest and right upper quadrant, a bifid, symmetric midline liver, and duplicated infrarenal inferior vena cavae (Fig. 5).

Genetic evaluation demonstrated heterozygous variants of uncertain significance in *CCDC39* (NM_181426.1:c.7 A>C) and *ZMYND10* (NM_015896.2:c.848G>A). Both genes are associated with primary ciliary dyskinesia with autosomal recessive inheritance.

The patient expired at 4 weeks of age after redirection to comfort care due to multiple congenital anomalies and poor predicted neurodevelopmental outcome.

## Discussion

This case includes craniofacial, spinal, and CNS features similar to those in previously described cases of duplicated pituitary gland plus syndrome, with additional findings of persistent falcine sinus, a severe hindbrain malformation involving the cerebellum, and multiple thoracoabdominal anomalies that have not been reported. The etiopathogenesis of duplicated pituitary gland (plus syndrome), including genetic causes, remains incompletely understood. Various theories include (1) aberrant early ventral midline development involving the axial mesoderm (embryonic tissue which gives rise to the prechordal plate and notochord--i.e., notochordal splitting); (2) incomplete twinning; (3) teratogenesis; and (4) extreme craniofacial clefting [1, 2].

With its newly described thoracoabdominal abnormalities, this case provides further support for the theory of duplication of the ventral midline structures. Duplicated pituitary gland plus syndrome may be a “ciliopathy,” with brain malformations overlapping with Joubert syndrome and craniofacial anomalies which have been described in numerous ciliopathies [1, 3]. Manifestations may result from abnormal primary ciliary function leading to excessive Sonic Hedgehog (SHH) expression and duplication of axial mesoderm-derived structures (e.g., the prechordal plate and notochord).

**Fig. 6:**
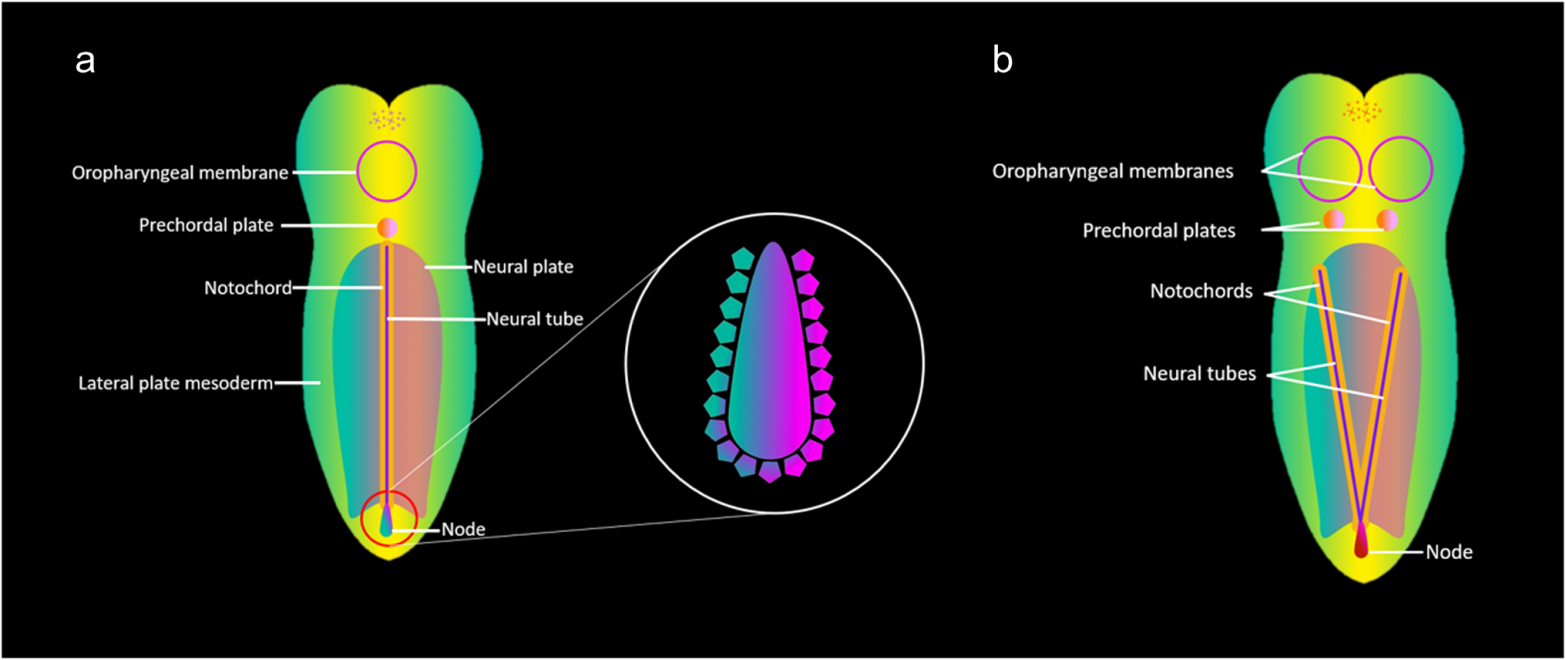
Diagrammatic explanation of possible etiopathogenesis in duplicated pituitary gland plus syndrome. The notochord and rostral prechordal plate, which are derived from axial mesoderm, are closely associated with a structure referred to as the “primitive node,” which contains the “left-right organizer” [2, 4, 5]. The normal configuration is diagrammed in part (**a**). The node is a ciliated structure that expresses chemical signals along a gradient (including Sonic Hedgehog (SHH)) to differentiate left- and right-sided structures in the developing embryo. A leading theory is that duplicated pituitary gland plus syndrome is a disorder affecting cilia (“ciliopathy”), with resultant abnormal expression of *SHH* and duplication of the prechordal plate and notochord (**b**). This leads to anomalies of numerous head, neck, spine, and CNS structures in the midline and parasagittal axes

The notochord plays a key role in patterning the embryo early in embryogenesis, acting through various chemical signaling pathways including the *SHH* pathway [5]. It is closely related to a structure called the “primitive node,” which contains a ciliated structure called the “left-right organizer.” Directional beating of ciliated cells in the node leads to expression of chemical signals along a gradient to differentiate left- and right-sided structures in the embryo [4]. Abnormal duplication of the prechordal plate, notochord, and node may lead to typical features of duplicated pituitary gland plus syndrome and disruption of normal left-right asymmetry [6]. A diagrammatic summary of potential etiopathogenesis is included in Fig. 6.

In our case, we propose that duplication of the oropharyngeal membrane and prechordal plate may have resulted in pituitary and infundibular duplication, skull base, nasal, and oral anomalies, and vascular anomalies including non-fused basilar arteries and persistent falcine sinus. Neural tube and plate (whose development is influenced by notochordal signaling) maldevelopment may have caused “prominent hypothalamic midline,” diencephalic-mesencephalic junction abnormality, hindbrain malformation, “limited ventral myeloschisis,” and ventral vertebral clefting [1]. The newly reported thoracoabdominal findings repeat the themes of abnormal symmetry and duplication, with abnormal left-right organization and anomalies of normally asymmetric body structures. Those in our case include horseshoe-type extralobar sequestration, symmetric-type liver, and splenic ectopia in a distribution dissimilar from that in typical polysplenia or left-sided isomerism, with two ectopic contralateral splenic sites unrelated to the dorsal mesogastrium of the left-sided stomach. The findings in this case and their hypothesized relationship to embryonic structure of origin are summarized in Table 1.

**Table 1:** Summary of findings categorized by possible embryonic structure of origin under the etiopathogenetic theory of notochordal splitting

Possible embryonic structure(s) of origin	Anomaly observed in current case
Prechordal plate, oropharyngeal membrane	1. Duplicated pituitary gland, infundibulum, and sella 2. Non-fused, paired basilar arteries 3. Persistent falcine sinus 4. Skull base anomalies 5. Hypertelorism 6. Nasal anomalies 7. Mass-like orofacial abnormality with disorganized mandibular elements 8. Bifid tongue
Neural tube and plate	1. “Prominent hypothalamic midline” 2. Diencephalic-mesencephalic junction dysplasia 3. *Hindbrain malformation involving the cerebellum* 4. “Limited ventral myeloschisis” 5. Anterior vertebral body “zipper-like” clefts 6. Vertebral formation/segmentation anomalies
Unknown cause, possibly related to severe notochordal splitting with disruption of left-right organizer in the primitive node	1. *“Horseshoe” extralobar pulmonary sequestration* 2. *Symmetric-type liver* 3. *Splenic ectopia with distribution unlike in polysplenia: two ectopic contralateral sites unrelated to greater curvature of the stomach* 4. *Duplicated inferior vena cava*

Newly described features observed in this case are italicized

The role of the genetic variants of uncertain significance was felt to be unclear. Interestingly, both the *CCDC39* and *ZMYND10* genes are associated with ciliary function. The *CCDC39* gene product is involved in the assembly of the dynein regulatory complex and inner dynein arms, which are important for normal ciliary motility [7]. The ZMYND10 protein is responsible for the correct localization of dynein arms, which is important for the formation and maintenance of cilia [8]. Notably, ciliary abnormalities are a feature of primary ciliary dyskinesia (Kartagener syndrome) which is associated with pathogenic variants in both *CCDC39* and *ZMYND10*. Features include situs inversus totalis [8]. While neither variant can be considered disease-causing in isolation, it is possible that the presence of missense variants in both genes in this case was a contributing factor. For instance, an increased burden of rare missense variants in these genes may increase the risk for this phenotype or play a role in a more complex, multigenic inheritance pattern. Ultimately, it is difficult to determine if these variants have any role in the disease. Research into the genetic mechanisms is needed.

## Conclusion

Although the cause remains unknown, we hypothesize that this severe case of duplicated pituitary gland plus syndrome with thoracoabdominal anomalies may be the result of a ciliopathy leading to elevated SHH expression and axial mesoderm duplication. Based on this example, clinicians may consider imaging of the chest and abdomen in patients with duplicated pituitary gland when identification of thoracoabdominal anomalies could influence additional diagnostic testing, clinical care, or prognostication.

## Data Availability

No datasets were generated or analysed during the current study.
